# Comparative Assessment of Cap Geometry in Small Incision Lenticule Extraction (SMILE) Using VisuMax 500 (500 kHz) and VisuMax 800 (2,000 kHz) and Its Association With Clinical Outcomes

**DOI:** 10.7759/cureus.113420

**Published:** 2026-07-26

**Authors:** Bu Ki Kim

**Affiliations:** 1 Ophthalmology, Onnuri Smile Eye Clinic, Seoul, KOR

**Keywords:** anterior segment optical coherence tomography, cap thickness, smile, uniformity, visumax 800

## Abstract

Background: In small incision lenticule extraction (SMILE), cap geometry can vary depending on the specific laser platform employed. Because differences in laser delivery speed between the VisuMax 500 (500 kHz) and VisuMax 800 (2,000 kHz) alter the duration of corneal applanation, variations in intraoperative stability and subsequent cap geometry may occur. To address this, this study compared corneal cap geometry between the VisuMax 500 and VisuMax 800 platforms and evaluated its associations with postoperative clinical outcomes.

Methods: This retrospective comparative study included eyes that underwent SMILE using either the VisuMax 500 or 800 system. One month postoperatively, corneal cap thickness was measured at nine horizontal points using anterior-segment optical coherence tomography. Cap accuracy was defined as the difference between achieved and intended thickness. Cap uniformity was quantified using the intra-subject standard deviation (SD) across the central 2.0-mm zone (central uniformity) and the full 6.0-mm zone (total uniformity). Correlations between uniformity and clinical outcomes were analyzed.

Results: No significant differences were observed in achieved cap thickness, cap accuracy, visual acuity, refraction, or higher-order aberrations between groups. However, the VisuMax 800 group demonstrated significantly superior central uniformity, with a significantly lower intra-subject SD (2.5 ± 1.2 µm) compared with the VisuMax 500 group (3.5 ± 1.6 µm; p = 0.010). In exploratory correlation analyses within the VisuMax 800 group, total uniformity correlated with postoperative sphere, spherical equivalent, and changes in spherical aberration, while central uniformity associated with changes in other higher-order aberrations; no such associations reached significance in the VisuMax 500 group.

Conclusions: The VisuMax 800 provides significantly improved central cap uniformity compared with the VisuMax 500 while maintaining similar cap accuracy and visual outcomes. These exploratory findings indicate that the VisuMax 800 platform is associated with greater central geometric consistency during cap creation, though observed correlations should be considered hypothesis-generating and require future prospective confirmation.

## Introduction

Small incision lenticule extraction (SMILE) has gained global popularity as an effective surgical modality for correcting refractive errors, supported by favorable clinical outcomes [1,2]. Unlike laser-assisted in situ keratomileusis (LASIK) or photorefractive keratectomy (PRK), where excimer-laser ablation using flying-spot scanning systems reshapes the corneal stroma, SMILE creates a refractive lenticule entirely with femtosecond (FS) photodisruption, followed by lenticule removal through a small incision [3]. During the lenticule-creation phase, the cornea is stabilized under suction, inducing transient structural deformation; consequently, the postoperative corneal geometry may vary depending on the specific laser platform and its associated energy delivery characteristics [4].

While the precision of flap creation in FS-LASIK is well known to influence postoperative optical quality, with superior thickness predictability and minimal variability leading to improved astigmatic neutrality and reduced higher-order aberrations (HOAs) [5,6], investigations evaluating the impact of cap geometry on clinical outcomes in SMILE remain limited. Although some studies suggest that greater cap uniformity is associated with better visual performance, these findings are based exclusively on the VisuMax 500 FS laser system (Carl Zeiss Meditec, Jena, Germany), which operates at a pulse repetition rate of 500 kHz [6,7]. The recently introduced VisuMax 800 platform operates at a markedly higher repetition rate of 2,000 kHz and incorporates several technological refinements, including reduced laser-on time, centration guidance, and cyclotorsion control [8]. Although the contact glass curvature and lenticule geometry are identical between the two systems, the substantially faster pulse delivery minimizes the duration of corneal applanation, potentially contributing to improved consistency in postoperative cap geometry.

Given this laser speed difference and the limited comparative data available, a direct evaluation of cap geometry between the VisuMax 500 and VisuMax 800 systems is warranted. The primary objective of this study was to compare corneal cap geometry, specifically central cap uniformity as the primary outcome, between the VisuMax 500 and VisuMax 800 platforms in SMILE. Secondarily, we explored the correlations between cap geometry and clinical outcomes within each platform group, noting that no direct overall differences in visual or refractive outcomes were observed between the two systems.

## Materials and methods

Study population

This was a retrospective, comparative study that included patients with myopia or myopic astigmatism who underwent SMILE using VisuMax 500 (the VisuMax 500 group) or VisuMax 800 (the VisuMax 800 group) at the Onnuri Smile Eye Clinic, Seoul, Republic of Korea, from June 2025 to August 2025. Patient allocation to either platform was based on equipment availability, surgical scheduling, and patient choice after thorough consultation, without specific clinical selection bias. Inclusion criteria included age of 19 years or older, corrected distance visual acuity (CDVA) of 20/30 or better, myopia from -0.50 to -7.00 diopters (D), astigmatism up to -4.50 D, stable refraction over two years, central corneal thickness (CCT) greater than 480 µm, and calculated postoperative residual bed thickness greater than 300 µm. Exclusion criteria included active or residual ocular disease, prior history of ocular surgery or trauma, and topographic evidence of keratoconus. Records were included if the patients had completed one month of postoperative follow-up. Because bilateral ocular measurements are more alike between fellow eyes than between eyes of different patients, and measurements from fellow eyes cannot be treated as if they were independent [9], only the right eyes were included for analysis. The study protocol was approved by the Public Institutional Review Board of the Ministry of Health and Welfare, Korea (P01-202606-01-041). All participants were thoroughly notified about the potential surgical complications, and informed consent was waived due to the retrospective design of the study. This study was conducted in accordance with the tenets of the Declaration of Helsinki.

Preoperative and clinical assessments

All patients underwent comprehensive preoperative and one-month postoperative ophthalmic evaluations. Uncorrected distance visual acuity (UDVA) and CDVA were measured using a standardized Snellen chart and converted to the logarithm of the minimum angle of resolution (logMAR) for statistical comparisons. Manifest and cycloplegic refractions were evaluated using subjective refraction. Corneal topography, CCT, and corneal HOAs across a 6.0-mm pupil diameter were measured using a Dual-Scheimpflug analyzer (Galilei G4, Ziemer Ophthalmic Systems AG, Port, Switzerland).

Surgical protocol

In all cases, the procedural target was emmetropia, and the same surgeon (KBK) performed the surgery. All procedures were performed under topical anesthesia using 0.5% proparacaine hydrochloride. A recommended nomogram, which included a 10% enhancement to prevent undercorrection, was applied to correct the spherical refractive error in both groups [4].

SMILE was performed using the VisuMax 500 or VisuMax 800 platforms in their respective groups.

In both groups, laser parameters were set to a pulse energy of 140 nJ and a spot spacing of 3.5 µm. The target cap thickness was intended to be 120 µm, and a 2.0-mm side-cut incision was created at the 11 o'clock position. The lenticule diameter ranged from 6.0 to 6.8 mm, with the cap diameter programmed to be 0.8 mm larger than the lenticule diameter in all cases. In the VisuMax 500 group, centration was achieved using the tear-film mark technique with patient fixation on the internal target. In the VisuMax 800 group, the surgeon used a joystick to align the displayed vertex reference point with the intended surgical center, and suction was initiated once the distance between the two points reached ≤ 0.1 mm. Lenticule extraction and postoperative management were identical in both groups and performed as previously described [4]. Postoperative topical management consisted of 0.5% moxifloxacin (Vigamox; Novartis, Basel, Switzerland) applied four times daily for five days, 0.1% fluorometholone (Fumelon; Hanmi Pharmaceutical, Seoul, Korea) used six times daily for the first five days and gradually tapered over four weeks, and preservative-free sodium hyaluronate artificial tears (Kynex; Alcon Korea, Seoul, Korea) applied for at least four weeks.

Anterior-segment optical coherence tomography (AS-OCT) measurement

**Figure 1:**
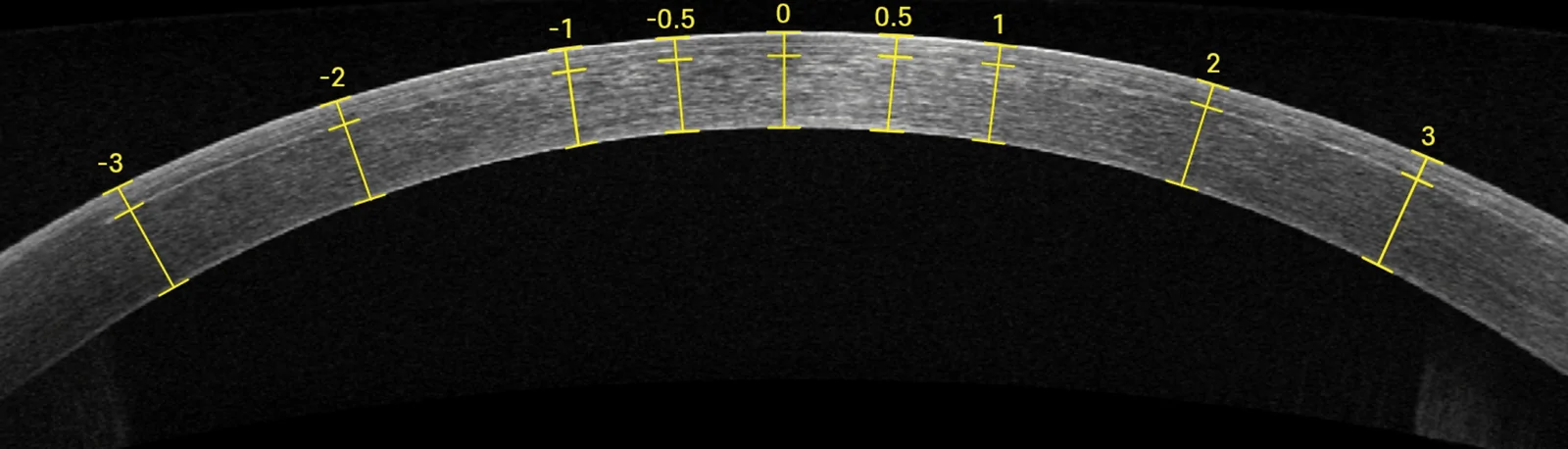
Horizontal anterior-segment optical coherence tomography image of the cornea after small incision lenticule extraction, illustrating the nine points used for cap thickness measurements. Measurements were obtained at the center (0) and at 0.5, 1.0, 2.0, and 3.0 mm from the center; negative and positive values represent the nasal and temporal locations, respectively.

All eyes were imaged at one month postoperatively using the Visante OCT (Carl Zeiss Meditec, Jena, Germany). The cornea was scanned horizontally along the central meridian, and corneal cap thickness was measured at nine points: the center and 0.5 mm, 1.0 mm, 2.0 mm, and 3.0 mm nasally and temporally of the center (Figure 1). All measurements were performed by a single masked examiner (KJA). To establish intra-observer repeatability, 15 randomly selected eyes were measured twice in a masked manner on separate days; the intra-class correlation coefficient was > 0.94 for all measurement points, demonstrating excellent repeatability. Cap accuracy was calculated as the signed difference between the achieved cap thickness and the intended cap thickness (achieved thickness: 120 µm).

Statistical analysis

Sample size calculation was performed prior to data collection using G*Power software (version 3.1.9.4, Heinrich Heine University, Düsseldorf, Germany). Based on preliminary data for central cap uniformity, an effect size of 0.85 was assumed. With an α level of 0.05 and an allocation ratio N2/N1 of 1.0, a total sample size of 52 eyes (26 eyes per group) was required to achieve a statistical power of 85% using a two-tailed independent t-test. Thus, the final sample size of 60 eyes (30 eyes per group) provided sufficient statistical power. Statistical analysis was performed using IBM SPSS Statistics for Windows, Version 25.0 (Released 2017; IBM Corp., Armonk, NY, USA). All data were subjected to the Kolmogorov-Smirnov test and were found to be normally distributed. The values are expressed as means ± standard deviation (SD). Between-group comparisons were performed using independent t-tests or chi-square tests, as appropriate. Pearson correlation analysis was utilized to evaluate the associations between cap uniformity and clinical parameters. Cap uniformity was assessed using the intra-subject SD across nine horizontal points, specifically categorized into central uniformity (central 2.0-mm zone) and total uniformity (full 6.0-mm zone). To mitigate the risk of type I errors arising from multiple exploratory correlation analyses, p-values from correlation matrices were reviewed with caution, and correlation strength was interpreted alongside clinical plausibility. Furthermore, exploratory correlation analysis was conducted to generate hypotheses rather than to establish direct platform superiority in clinical outcomes, given that overall postoperative clinical outcomes did not differ significantly between groups. A p-value ≤ 0.05 was considered statistically significant.

## Results

The VisuMax 500 and VisuMax 800 groups each included 30 eyes. There were no significant differences between the two groups in age, refractive errors, CCT, optic zone diameter, keratometry, or corneal HOAs (Table 1). No intraoperative or postoperative complications were observed in either group throughout the follow-up period.

**Table 1:** Patients’ demographics. IOP: intraocular pressure, CCT: central corneal thickness, D: diopters, SE: spherical equivalent, K: keratometry, HOAs: higher-order aberrations. ^*^Independent sample t-test. ^†^Chi-square test. For the chi-square test applied to sex, the test statistic was χ^2^ = 0.49, with a degree of freedom (df) = 1, and an effect size (Cramer's V) = 0.07.

	VisuMax 500 (n = 30)	VisuMax 800 (n = 30)	p-value
Age (years)	25.63 ± 6.66	23.70 ± 4.96	0.207*
Sex (female, n (%))	16 (53.3)	17 (56.7)	0.810^†^
IOP (mmHg)	19.80 ± 1.83	19.63 ± 1.71	0.717*
Pupil diameter (mm)	6.85 ± 0.62	6.97 ± 0.73	0.496*
Optic zone diameter (mm)	6.54 ± 0.20	6.53 ± 0.25	0.822*
CCT (µm)	569.73 ± 26.44	565.93 ± 34.21	0.632*
Refractive errors (D)			
Spherical	-3.35 ± 1.40	-3.23 ± 1.46	0.753*
Cylindrical	-0.94 ± 0.69	-1.38 ± 1.24	0.094*
SE	-3.70 ± 1.47	-3.93 ± 1.61	0.580*
Flat K (D)	41.95 ± 1.68	42.41 ± 1.64	0.290*
Steep K (D)	43.24 ± 1.71	44.03 ± 1.77	0.083*
Corneal HOAs			
Oblique trefoil (C_3_^-3^)	-0.01 ± 0.13	0.02 ± 0.14	0.527*
Vertical coma (C_3_^-1^)	0.06 ± 0.21	0.11 ± 0.16	0.356*
Spherical aberration (C_4_^0^)	0.18 ± 0.07	0.18 ± 0.08	0.661*
Horizontal coma (C_3_^+1^)	-0.10 ± 0.14	-0.13 ± 0.18	0.477*
Vertical trefoil (C_3_^+3^)	-0.06 ± 0.09	-0.06 ± 0.16	0.976*
Total corneal HOAs	1.41 ± 0.61	1.79 ± 0.86	0.052*
Other HOAs	0.16 ± 0.08	0.18 ± 0.12	0.351*

Cap morphology

The achieved cap thickness at each measurement point is compared in Table 2. In both groups, the thinnest cap was observed at the center, measuring 123.5 ± 5.6 μm and 122.6 ± 4.4 μm for the VisuMax 500 and VisuMax 800 groups, respectively, and cap thickness increased progressively toward the peripheral points. There were no significant differences in achieved cap thickness between the groups at any point.

**Table 2:** Comparison of corneal cap thickness and uniformity between the VisuMax 500 and VisuMax 800 groups. 0 indicates the corneal center; negative and positive values represent the nasal and temporal distances (mm) from the center, respectively, along the horizontal meridian. Statistical analysis was performed using the independent t-test. ^*^p < 0.05.

	-3	-2	-1	-0.5	0	0.5	1	2	3	Total uniformity (SD)	Central uniformity (SD)
VisuMax 500 (µm)	131.7 ± 8.4	128.0 ± 6.2	125.0 ± 4.4	124.4 ± 5.0	123.5 ± 5.6	123.6 ± 6.1	124.6 ± 5.3	124.9 ± 6.4	128.5 ± 9.5	6.0 ± 1.7	3.5 ± 1.6
VisuMax 800 (µm)	132.4 ± 4.9	127.7 ± 4.9	124.3 ± 4.3	123.2 ± 3.8	122.6 ± 4.4	122.6 ± 4.5	123.7 ± 4.6	125.0 ± 5.3	129.4 ± 7.2	5.1 ± 1.7	2.5 ± 1.2
p-value	0.724	0.818	0.572	0.331	0.054	0.44	0.499	0.983	0.67	0.052	0.010^*^

Cap accuracy

Table 3 presents the differences between the achieved and intended cap thickness values. In both groups, the smallest deviation was observed at the central point, measuring 4.9 ± 4.4 μm and 3.1 ± 2.7 μm for the VisuMax 500 and VisuMax 800 groups, respectively, with deviations increasing toward the periphery. No significant differences were found between the two groups at any measurement point.

**Table 3:** Comparison of cap thickness deviations (achieved vs. intended) between the two groups. Statistical analysis was performed using the independent t-test.

	-3	-2	-1	-0.5	0	0.5	1	2	3
VisuMax 500 (µm)	12.7 ± 6.9	9.0 ± 4.6	5.4 ± 3.8	5.3 ± 4.0	4.9 ± 4.4	4.9 ± 5.0	5.4 ± 4.4	6.4 ± 4.9	10.9 ± 6.5
VisuMax 800 (µm)	12.6 ± 4.4	7.8 ± 4.7	4.8 ± 3.7	4.1 ± 2.8	3.1 ± 2.7	3.4 ± 3.8	4.6 ± 3.7	5.7 ± 4.5	10.3 ± 5.8
p-value	0.947	0.319	0.564	0.195	0.066	0.187	0.449	0.567	0.709

Cap uniformity

While the total uniformity did not reach statistical significance between the groups (5.1 ± 1.7 μm for VisuMax 800 vs. 6.0 ± 1.7 μm for VisuMax 500; p = 0.052), central uniformity was significantly superior in the VisuMax 800 group (2.5 ± 1.2 μm) compared to the VisuMax 500 group (3.5 ± 1.6 μm; p = 0.010; Table 2). This suggests that the VisuMax 800 platform may produce a more uniform cap, particularly within the central corneal region.

Clinical outcomes and their correlations with cap uniformity

Table 4 summarizes the clinical outcomes of the two groups. No significant differences were observed between the VisuMax 500 and VisuMax 800 groups in terms of UDVA, CDVA, residual refractive errors, or corneal HOAs (all p > 0.05).

**Table 4:** Comparisons of clinical outcomes at one month postoperatively between the VisuMax 500 and VisuMax 800 groups. UDVA: uncorrected distance visual acuity, logMAR: logarithm of the minimum angle of resolution, CDVA: corrected distance visual acuity, D: diopters, SE: spherical equivalent, HOAs: higher-order aberrations. Statistical analysis was performed using the independent t-test.

	VisuMax 500	VisuMax 800	p-value
UDVA (logMAR)	-0.05 ± 0.04	-0.06 ± 0.04	0.237
CDVA (logMAR)	-0.06 ± 0.03	-0.06 ± 0.04	0.871
Refractive errors (D)			
Spherical	-0.03 ± 0.38	-0.08 ± 0.37	0.607
Cylindrical	-0.32 ± 0.21	-0.34 ± 0.21	0.646
SE	-0.19 ± 0.35	-0.25 ± 0.35	0.492
Corneal HOAs (µm)			
Oblique trefoil (C_3_^-3^)	0.07 ± 0.21	0.03 ± 0.20	0.493
Vertical coma (C_3_^-1^)	-0.05 ± 0.23	0.02 ± 0.19	0.219
Spherical aberration (C_4_^0^)	0.29 ± 0.15	0.25 ± 0.15	0.335
Horizontal coma (C_3_^+1^)	-0.21 ± 0.18	-0.19 ± 0.19	0.787
Vertical trefoil (C_3_^+3^)	-0.09 ± 0.14	-0.02 ± 0.17	0.082
Total corneal HOAs	1.86 ± 0.42	1.93 ± 0.50	0.575
Other HOAs	0.24 ± 0.10	0.26 ± 0.10	0.355

Table 5 presents the correlations between total/central uniformity and visual performance metrics, including changes in HOAs, visual acuity, and residual refractive errors. Pearson correlation analysis showed that in the VisuMax 500 group, neither total uniformity nor central uniformity was significantly associated with any of the evaluated visual performance metrics. In contrast, the VisuMax 800 group showed several significant correlations. Total uniformity exhibited a significant negative correlation with the change in spherical aberration (SA) (r = -0.377, p = 0.040). Additionally, total uniformity showed significant positive correlations with postoperative sphere (r = 0.480, p = 0.007) and spherical equivalent (SE) (r = 0.478, p = 0.008). Furthermore, central uniformity in the VisuMax 800 group was significantly and positively correlated with the change in other HOAs (r = 0.372, p = 0.043).

**Table 5:** Correlation between cap uniformity and clinical outcomes. OT: oblique trefoil, VC: vertical coma, SA: spherical aberration, HC: horizontal coma, VT: vertical trefoil, HOAs: higher-order aberrations, UDVA: uncorrected distance visual acuity, CDVA: corrected distance visual acuity, SE: spherical equivalent. Statistical analysis was performed using the Pearson correlation analysis. ^*^p < 0.05.

Group	Cap uniformity	Variable	r (Pearson)	p-value
VisuMax 500	Total uniformity	Change in OT	0.104	0.585
		Change in VC	0.271	0.147
		Change in SA	-0.038	0.844
		Change in HC	0.099	0.601
		Change in VT	0.005	0.98
		Change in total corneal HOAs	0.027	0.887
		Change in other HOAs	0.238	0.206
		Postoperative UDVA	-0.05	0.795
		Postoperative CDVA	0.029	0.969
		Postoperative sphere	-0.184	0.33
		Postoperative cylinder	-0.01	0.96
		Postoperative SE	-0.199	0.291
	Central uniformity	Change in OT	0.142	0.453
		Change in VC	0.32	0.084
		Change in SA	-0.016	0.934
		Change in HC	-0.108	0.569
		Change in VT	-0.096	0.613
		Change in total corneal HOAs	0.214	0.257
		Change in other HOAs	0.285	0.127
		Postoperative UDVA	0.073	0.7
		Postoperative CDVA	0.021	0.874
		Postoperative sphere	0.04	0.834
		Postoperative cylinder	0.098	0.606
		Postoperative SE	0.072	0.707
VisuMax 800	Total uniformity	Change in OT	-0.076	0.688
		Change in VC	0.047	0.805
		Change in SA	-0.377	0.040*
		Change in HC	0.21	0.265
		Change in VT	0.201	0.287
		Change in total corneal HOAs	-0.285	0.127
		Change in other HOAs	-0.261	0.164
		Postoperative UDVA	-0.011	0.955
		Postoperative CDVA	-0.008	0.987
		Postoperative sphere	0.48	0.007*
		Postoperative cylinder	-0.121	0.526
		Postoperative SE	0.478	0.008*
	Central uniformity	Change in OT	0.261	0.164
		Change in VC	0.043	0.823
		Change in SA	-0.343	0.064
		Change in HC	0.071	0.71
		Change in VT	-0.028	0.883
		Change in total corneal HOAs	0.024	0.898
		Change in other HOAs	0.372	0.043*
		Postoperative UDVA	-0.02	0.916
		Postoperative CDVA	0.012	0.959
		Postoperative sphere	-0.03	0.876
		Postoperative cylinder	-0.323	0.082
		Postoperative SE	-0.131	0.492

## Discussion

This study provides a comparative assessment of postoperative cap geometry between the VisuMax 500 and VisuMax 800 FS laser platforms. Although both systems produced comparable achieved cap thickness and cap accuracy, the VisuMax 800 demonstrated significantly better cap uniformity within the central 2.0-mm zone. Notably, while exploratory correlation analyses in the VisuMax 800 group showed associations between central uniformity and other HOAs, and between total uniformity and SA/sphere/SE, these correlations were not observed in the VisuMax 500 group. However, because formal interaction tests or direct coefficient comparisons between the two platforms were not performed, these findings do not establish a definitive platform-specific relationship. Consequently, these observations should be interpreted with caution as exploratory and hypothesis-generating findings rather than proof of clinical outcome superiority.

Cap creation in SMILE is influenced by a combination of biomechanical responses and laser-tissue interaction dynamics, resulting in measurable deviations between intended and achieved stromal dissection depth [6,7]. Prior analyses of cap morphology after SMILE have shown that the achieved cap thickness typically differs from the intended value by approximately ±5 µm at the central zone and up to ±10 µm toward the periphery [10]. These deviations reflect the intrinsic geometry of the lamellar cut and suction-related corneal deformation; while central regions generally maintain closer alignment to the programmed depth, peripheral regions show progressively larger discrepancies [10]. This variability has been attributed to transient stromal displacement during lamellar scanning, differences in pulse frequency and scanning trajectory across platforms, and the interaction of femtosecond energy with the corneal microstructure [11]. Cap morphology has also been linked to optical quality, as deviations in uniformity correlate with increased intraocular scattering and changes in HOAs [10]. Furthermore, studies have demonstrated that even modest variability in lamellar thickness can influence refractive outcomes, emphasizing the clinical relevance of achieving a stable and uniform dissection plane [12]. Collectively, these observations suggest that cap formation in SMILE is determined not only by preset parameters but also by platform-specific laser characteristics and the biomechanical behavior of the cornea during suction and scanning.

In the present study, both platforms demonstrated high cap accuracy, with minimal central deviation and a gradual increase toward the periphery. This pattern is consistent with previous studies on cap morphology in SMILE, which have uniformly reported thinner central caps and progressive peripheral thickening [6,7]. This reflects the inherent geometry of lenticule dissection, characterized by central uniformity and a peripheral increase in thickness variation [13]. However, while cap accuracy did not differ between groups, the VisuMax 800 exhibited significantly superior central uniformity compared to the VisuMax 500. Importantly, this difference represented an approximately 29% reduction in intra-subject variability, suggesting a meaningful improvement in the consistency of central stromal modification. The central 2.0-mm zone may be particularly relevant because it corresponds to the region with the greatest contribution to retinal image formation. Unlike peripheral cap variations, subtle irregularities within the central optical zone have a disproportionately larger influence on wavefront propagation and postoperative optical quality. Therefore, central uniformity may represent a more clinically meaningful indicator of surgical precision than overall cap thickness accuracy alone. This aligns with the theoretical advantages of higher-frequency laser delivery, where faster scanning reduces the temporal interval between laser spots, promoting a smoother and more continuous dissection plane [14]. The significantly reduced "laser-on time" with the VisuMax 800 minimizes the duration of corneal applanation and suction. This abbreviation of the procedure may mitigate transient mechanical deformation of the cornea and reduce the window for potential micro-movements or suction instability. Furthermore, the VisuMax 800 platform incorporates an active centration guidance system, ensuring the surgical center aligns within ≤ 0.1 mm of the visual axis. This high-precision centration, combined with rapid pulse delivery, may also contribute to the superior central uniformity observed, as minimal decentration is critical for maintaining uniformity within the central optical zone. Given that the central optical zone carries the highest importance in image formation [15,16], the improved central uniformity of the VisuMax 800 may directly enhance postoperative optical quality, although this hypothesis warrants further validation through patient-reported outcomes. However, given the retrospective design of this study, proposed mechanisms involving reduced applanation time or enhanced suction stability remain speculative and require prospective validation.

In the present study, although no significant differences were observed between the two groups in UDVA, CDVA, postoperative refractive errors, or postoperative corneal HOAs, only the VisuMax 800 group showed significant correlations between cap geometry and clinical metrics. Specifically, central uniformity was associated with changes in other HOAs, while total uniformity correlated with SA and postoperative sphere/SE. In contrast, no significant associations were identified between cap uniformity and visual performance metrics in the VisuMax 500 group. One possible explanation for this discrepancy in correlation profiles between the two platforms is the influence of intraoperative factors inherent to lower-frequency laser delivery. In the VisuMax 500 group, the prolonged laser-on time and extended suction duration may induce cumulative mechanical tissue deformation and subtle micro-movements, creating a baseline of optical noise that masks any systematic association between macro-geometric uniformity and visual outcomes. Conversely, the ultra-high-frequency and rapid scanning of the VisuMax 800 may mitigate these transient corneal alterations. By effectively suppressing intraoperative micro-irregularities and stabilization artifacts, the VisuMax 800 platform may allow potential associations between postoperative cap geometry and optical characteristics to become more readily detectable. These findings suggest that the relationship between cap geometry and postoperative optical parameters may be more readily detectable when geometric variability is reduced, although this remains speculative and warrants further investigation. Nevertheless, because these correlation analyses were exploratory, lacked formal multiplicity corrections, and were not accompanied by formal statistical tests comparing correlation coefficients between platforms, these results cannot establish a true platform-specific difference in optical performance and must be viewed strictly as preliminary observations.

The present study has several limitations that warrant consideration. First, the retrospective design and relatively small sample size may limit the generalizability of the findings, although G*Power calculations confirmed adequate statistical power for the primary outcome. Patient allocation to each platform was determined based on scheduling and equipment availability, which introduces potential selection, temporal, or learning-curve biases. However, because the operating surgeon was highly experienced with both platforms prior to the study period, learning-curve effects were likely minimal. Second, corneal cap geometry was evaluated using a time-domain AS-OCT system along a single horizontal cross-sectional meridian. Although this approach allows precise relative comparison across matched groups and benefits from high intra-observer repeatability (ICC > 0.94), time-domain OCT offers lower axial resolution than newer spectral-domain or swept-source OCT platforms. Furthermore, a single horizontal scan cannot fully capture three-dimensional volumetric cap architecture or asymmetrical regional variations. Third, cap geometry was evaluated at one month postoperatively. Although early corneal healing is particularly active during this period, extended follow-up is required to evaluate long-term cap stability amidst ongoing stromal remodeling. Fourth, corneal biomechanical parameters (e.g., corneal hysteresis) were not measured directly. Correlating geometric findings with direct biomechanical metrics in future prospective studies would provide a deeper understanding of laser-tissue interaction. Finally, multiple exploratory correlation analyses were performed without formal multiplicity correction (e.g., Bonferroni correction), which increases the risk of type I error. Moreover, because formal statistical interaction tests or direct comparisons of correlation coefficients (e.g., Fisher’s z-transformation) between the two platforms were not performed, a statistically significant correlation observed in the VisuMax 800 group alone does not definitively establish a true platform difference. Therefore, these correlation results should be interpreted cautiously as hypothesis-generating observations rather than definitive evidence of clinical outcome superiority.

## Conclusions

In conclusion, the VisuMax 800 produces significantly greater central cap uniformity compared with the VisuMax 500 while maintaining comparable overall cap accuracy and clinical outcomes. Although exploratory correlation analyses revealed associations between cap uniformity and specific optical and refractive metrics in the VisuMax 800 group, these preliminary findings should be interpreted cautiously as hypothesis-generating observations. Overall, the VisuMax 800 is associated with more consistent central corneal modification during SMILE, though the long-term clinical relevance of this geometric improvement requires prospective validation in larger cohorts.
